# ASSESSMENT OF ISOMETRIC AND ISOKINETIC ANKLE STRENGTH MEASURES: A PILOT STUDY

**DOI:** 10.1590/1413-785220253302e288925

**Published:** 2025-10-13

**Authors:** CAROLINA LINS, ANDREZA RIBEIRO BATISTA DE OLIVEIRA, MARINA SQUARIZI SIMÕES CHAGAS, FELIPPE RIBEIRO, ALBERTO CLIQUET, RODRIGO GONÇALVES PAGNANO

**Affiliations:** 1. Universidade Estadual de Campinas (UNICAMP), Faculdade de Ciencias Medicas, Departamento de Ortopedia, Reumatologia e Traumatologia, Campinas, Sao Paulo, SP, Brazil.; 2. Centro de Reabilitacao Fisica Monica Cristina Blanco, Hortolandia, Sao Paulo, SP, Brazil.; 3. Instituto Wilson Mello, Campinas, Sao Paulo, SP, Brazil.

**Keywords:** Ankle, Rehabilitation, Muscle Strength Dynamometer, Tornozelo, Reabilitação, Dinamômetro de Força Muscular

## Abstract

**Objective:**

To evaluate isometric and isokinetic ankle strength in of dorsiflexion (DF), plantar flexion (PF), inversion (INV), and eversion (EVE) in healthy individuals.

**Methods:**

A cross-sectional study was conducted with individuals aged 18 to 60 years, of both sexes. The Lafayette® isometric manual dynamometer was used to evaluate isometric strength, the Humac Norm® isokinetic dynamometer to evaluate isokinetic strength, and the IPAQ questionnaire (International Physical Activity Questionnaire) for the level of physical activity. Statistical analysis compared sex, dominance, and physical activity level with isometric and isokinetic strengths using the Spearman coefficient and the Mann-Whitney test.

**Results:**

There was a difference between genders for dominant and non-dominant limbs in isokinetic strength and not in isometric strength. There was a difference between isokinetic strength variables and physical activity levels. The isokinetic strength of dominant PF (p=0.0153), non-dominant (p=0.0287), and non-dominant INV (p=0.0183) demonstrated that very active individuals have a higher torque peak than irregularly active and sedentary individuals.

**Conclusion:**

The results demonstrated greater isokinetic strength in men than in women and active individuals compared to sedentary ones. However, it was not possible to establish an association between isometric and isokinetic ankle measurements. Level of Evidence IV, Cross-Sectional Study.

## RESUMO

**Objetivo:**

Avaliar forças isométricas e isocinéticas de dorsiflexão (DF), flexão plantar (FP), inversão (INV) e eversão (EVE) do tornozelo em indivíduos saudáveis.

**Métodos:**

Estudo transversal com indivíduos entre 18 e 60 anos, de ambos os sexos. Foram utilizados o dinamômetro manual isométrico Lafayette® para avaliar a força isométrica, o dinamômetro isocinético Humac Norm® para avaliar a força isocinética e o questionário IPAQ (Questionário Internacional de Atividade Física) para o nível de atividade física. A análise estatística comparou sexo, dominância e nível de atividade física com forças isométricas e isocinéticas por meio do coeficiente de Spearman e do teste de Mann-Whitney.

**Resultados:**

Houve diferença entre gêneros para membros dominantes e não dominantes nas forças isocinéticas e não na força isométrica. Houve diferença entre variáveis de força isocinética e níveis de atividade física. Força isocinética de FP dominante (p=0,0153), não dominante (p=0,0287) e INV não dominante (p=0,0183) demonstraram que indivíduos muito ativos apresentam pico de torque maior que os irregularmente ativos e sedentários.

**Conclusão:**

Os resultados demonstraram maior força isocinética em homens do que em mulheres, assim como em indivíduos ativos em comparação aos sedentários. No entanto, não foi possível estabelecer uma associação entre as medidas isométricas e isocinéticas do tornozelo. Nível de Evidência IV, Estudo Transversal.

## INTRODUCTION

Various treatment modalities are used in foot and ankle tendinopathies, both conservative and surgical, with the assessment of muscle strength (MS) being an important component in the rehabilitation process of these cases. Various methods are used to achieve this, such as manual strength testing, manual dynamometers and isokinetic dynamometers.^1,2^

The manual strength test is the most common, based on a subjective grading system, which consists of performing movement against resistance applied by the examiner or resistance against gravity applied during the test.^3,4^ The isokinetic dynamometer is the most accurate instrument for evaluating MS, however its disadvantages are the high cost of the equipment and the need for a qualified professional to carry out the test.^5^ The third method, isometric dynamometry, preserves the efficiency and adaptability of the manual strength test, promoting a more accurate and objective assessment of MS.^6^ Among the portable isometric dynamometry devices is the Lafayette® Hand-Held-Dynamometer. Researchers report the usefulness of manual dynamometers in rehabilitation services due to the benefit and objectivity for both the evaluator and the patient.

There are studies that compare the effectiveness, reproducibility and reliability of these devices in evaluating the knee and shoulder joints.^7,8^ However, there are few consistent studies on the application of manual dynamometers and there is no definitive protocol for evaluating isometric and isokinetic strength of the ankle joint in the sagittal and frontal plane. This study aims to evaluate isometric and isokinetic forces of dorsiflexion (DF), plantar flexion (PF), inversion (INV) and eversion (EVE) of the ankle in healthy individuals.

## MATERIALS AND METHODS

Cross-sectional study, approved by the FCM-Unicamp Research Ethics Committee, fulfilling the requirements of resolution 466/2012 CNS/MS and complementary requirements in the preparation of the protocol and in obtaining this Informed Consent Form. The recruitment and data collection process took place at the Universidade Estadual de Campinas (UNICAMP) and at an orthopedic clinic Wilson Mello Institute.

### Population

25 volunteers of both genders, aged between 18 and 60 years old, were selected. Initially, all individuals received explanations about the study and, upon agreeing, signed the free and informed consent form. Firstly, the subjects were interviewed to collect personal data, age, anthropometric data, dominance of the lower limbs, goniometry, investigation of previous lower limb dysfunctions and the IPAQ questionnaire for level of physical activity. The inclusion criteria were healthy female and male individuals, aged between 18 and 60 years, who agreed to participate in the study, signing the free and informed consent form. The exclusion criteria were subjects with previous lower limb injuries or surgeries or presenting severe or decompensated systemic diseases.

### Instruments and Procedures

#### Instruments

Participants answered the IPAQ physical activity questionnaire (short version) that assesses the individual’s daily physical capacity, classifying them as very active, active, irregularly active (A and B) and sedentary. Activities carried out at work, going from one place to another, leisure, sport, exercise or part of their activities at home or in the garden were considered.^9^

Goniometry assessed range of motion (ROM). For DF, the individual was positioned sitting, with the knees flexed at around 25º or 30º so as not to interfere with the action of the muscles in the posterior region of the thigh and ankle in a neutral position. The fixed arm of the goniometer was placed parallel to the lateral surface of the fibula and the movable arm parallel to the lateral surface of the fifth metatarsal, with the axis of the goniometer positioned next to the lateral malleolus. For analysis of PF ROM, the positioning was the same. In the assessment of INV and EVE, the individual’s positioning was like the previous ones, but with an axis between the talus and calcaneus, the arm fixed towards the anterior surface of the tibia and the mobile arm towards the third finger.^10^

To measure muscular strength, individuals were instructed about appropriate clothing, leaving their lower limbs free and barefoot. The patients underwent a 5-minute cardiocirculatory and musculoskeletal warm-up on a stationary bike (60-70 rpm) and were instructed on the procedures to be performed.

Isometric strength, with a unit of measurement in kilogram-force (kgf), was tested using the Lafayette Model 01165A isometric dynamometer (Lafayette Instrument, United States of America), size: 3.16” x 5.11” x 1.6” (8.03cm x 12.98 cm x 4.1cm), range: 0-300 lbs (136.1kg) (1335 N), accuracy: ± 1% of full scale or ± 0.2 lbs, resolution: 0.1lbs/0.1kg/0.1N(0-999.9N) /1N(1000N-1335N)).^11^

The patient was positioned in the supine position, with hip and knee flexion of approximately 30º and a neutral ankle position (90º),^12^ which was stabilized by Velcro straps. The dynamometer was positioned on the ventral surface of the metatarsal head to collect plantar flexion data, on the dorsal surface of the metatarsal head to collect DF data, just below the head of the medial surface of the 1st metatarsal for inversion, just below the head of the lateral surface of the 5th metatarsal for eversion. The device recorded peak force for a period of six seconds, providing a reliable, accurate and stable isometric muscle strength reading.^13^

Isokinetic strength was measured using a Humac Norm® brand isokinetic dynamometer (Computer Sports Medicine, inc; United States of America). To perform the maximum torque exercises, the dynamometer was positioned in the same way as the isometric dynamometer, in the supine position, with Velcro straps located on the distal third of the thigh and abdomen. The thigh stabilizing cushion was placed close to the knee joint and the hip and knee joints were positioned at 30° flexion. The ankle remained in a neutral position (90º).^11^

To avoid detection bias, the subjects underwent familiarization with the isokinetic test, that is, four warm-up repetitions at 90% of maximum strength. Then, the real test was done with a belt, repetitions at 100% of maximum force and both at a speed of 30°/s.

## Statistical Analisys

Exploratory data analysis was carried out using summary measures (mean, standard deviation, minimum, median, maximum, frequency and percentage). The correlation between numerical variables was assessed using the Spearman coefficient. Comparison between genders was performed using the Mann-Whitney test. The significance level adopted was 5%.

## RESULTS

The 25 volunteers, with an average age of 28.8 (± 9.1) years, were 11 women and 14 men, with an average weight of 73.6 (± 12.9) kilograms, height 173.2 (± 11 .2) centimeters and body mass index of 24.4 (± 3.1) kg/m2.

Table 1 presents the mean values and standard deviations of goniometry in dorsiflexion, plantar flexion, inversion and eversion movements of the volunteers’ dominant limbs.

**Table 1:** Mean values and standard deviations of goniometry of the volunteers’ dominant limbs.

Variables	Healthy Subjects n = 25	Female n = 11	Male n = 14
DF (degree)	8.0 ± 4.1	9.1 ± 4.1	7.1 ± 3.9
PF (degree)	21.0 ± 7.6	20.0 ± 5.1	21.8 ± 9.2
INV (degree)	27.2 ± 5.4	26.5 ± 4.2	27.8 ± 6.3
EVE (degree)	13.9 ± 5.1	12.6 ± 4.6	15.0 ± 5.4

DF = dorsiflexion; PF = plantar flexion; INV = inversion; EVE = eversion.

Table 2 presents the mean values and standard deviations of goniometry in dorsiflexion, plantar flexion, inversion and eversion movements of the volunteers’ non-dominant limbs.

**Table 2:** Mean values and standard deviations of goniometry of the volunteers’ non-dominant limbs.

Variables	Healthy Subjects n = 25	Female n = 11	Male n = 14
DF (degree)	6.4 ± 3.9	7.6 ± 4.4	5.43 ± 3.2
PF (degree)	24.0 ± 7.1	23.0 ± 5.8	24.8 ± 8.1
INV (degree)	23.8 ± 7.0	25.8 ± 4.6	22.2 ± 8.6
EVE (degree)	16.6 ± 7.9	17.5 ± 7.5	15.8 ± 8.4

DF = dorsiflexion; PF = plantar flexion; INV = inversion; EVE = eversion.

Table 3 presents the mean values and standard deviations of the isometric strength of the dorsiflexion, plantar flexion, inversion and eversion movements of the volunteers’ dominant and non-dominant limbs.

**Table 3:** Mean values and standard deviations of the volunteers’ isometric strength.

Variables	Male (D) n = 14	Female (D) n = 11	p-values	Male (ND) n = 14	Female (ND) n = 11	p-value
DF (kgf)	18.6 ± 4.0	17.2 ± 7.0	0.1627	16.7 ± 3.3	14.4 ± 4.0	0.1469
PF (kgf)	21.8 ± 7.0	17.2 ± 5.0	0.0752	22.1 ± 8.6	16.5 ± 5.2	0.072
INV (kgf)	10.6 ± 4.3	10.1 ± 4.2	0.7843	12.2 ± 5.5	11.1 ± 4.2	0.8911
EVE (kgf)	13.1 ± 4.0	13.3 ± 6.2	0.5470	12.5 ± 3.9	12.1 ± 6.3	0.3380

D = dominant; ND = non dominant; DF = dorsiflexion; PF = plantar flexion; INV = inversion; EVE = eversion; kgf= kilogram-force.

Table 4 presents the mean values and standard deviations of the isokinetic strength of the dorsiflexion, plantar flexion, inversion and eversion movements of the volunteers’ dominant and non-dominant limbs.

**Table 4:** Mean values and standard deviations of the volunteers’ isokinetic strength.

Variables	Male (D) n = 14	Female (D) n = 11	p-value	Male (ND) n = 14	Female (ND) n = 11	p-value
DF (Nm)	52.9 ± 23.9	30.1 ± 8.7	0.0018*	48.9 ± 18.9	33.1 ± 12.1	0.0229*
PF (Nm)	130.2 ± 36.4	55.9 ± 23.5	0.0002*	123.5 ± 30.6	56.2 ± 17.6	<0.0001*
INV (Nm)	41.3 ± 7.8	25.1 ± 6.8	0.0002*	49.2 ± 11.3	30.1 ± 8.2	0.0006*
EVE (Nm)	27.7 ± 6.6	18.7 ± 5.9	0.0037*	26.8 ± 5.0	18.0 ± 3.9	0.0004*

D = dominant; ND = non dominant; DF = dorsiflexion; PF = plantar flexion; INV = inversion; EVE = eversion; Nm= Newton-meter; * = significance p <0,05.

Table 5 presents the average deficit values, in percentage, for dorsiflexion, plantar flexion, inversion and eversion movements.

**Table 5:** Average values of isometric and isokinetic deficits.

Variables	Male n = 14		Female n = 11	
	Isometric	Isokinetic	Isometric	Isokinetic
DF	14.1 ± 0.0	11.2 ± 10.1	21.4 ± 0.1	19.0 ± 20.9
PF	15.1 ± 0.1	16.5 ± 11.3	19.0 ± 0.1	13.3 ± 14.4
INV	13.9 ± 0.0	19.0 ± 11.5	15.1 ± 0.1	22.9 ± 10.6
EVE	11.3 ± 0.0	13.4 ± 10.5	16.3 ± 0.0	8.9 ± 8.3

DF = dorsiflexion; PF = plantar flexion; INV = inversion; EVE = eversion.

Table 6 presents the mean values and standard deviations of the isokinetic strength of the dorsiflexion, plantar flexion, inversion and eversion movements of the volunteers’ dominant and non-dominant limbs, associated with the IPAQ.

**Table 6:** Mean values and standard deviations of isokinetic strength and physical activity level of volunteers.

		IPAQ		
	Isokinetic Strength	Very Active	Irregularly Actives e Sedentaries	P-value
Dominant	DF	51.7 ± 23.8	42.2 ± 30.4	0.43
	FP	136.7 ± 27.1	65.8 ± 27.4	0.01*
	INV	41.7 ± 7.1	31.0 ± 11.1	0.10
	EVE	28.7 ± 7.5	19.5 ± 6.8	0.14
Non Dominant	DF	52.8 ± 23.0	42.0 ± 18.3	0.24
	FP	123.2 ± 23.1	69.1 ± 20.8	0.02*
	INV	50.5 ± 10.0	30.7 ± 9.5	0.01*
	EVE	25.4 ± 4.7	20.1 ± 6.2	0.20

IPAQ = questionnaire of level of activity; DF = dorsiflexion; PF = plantar flexion; INV = inversion; EVE = eversion; * = significance p <0,05.

**Figure 1:**
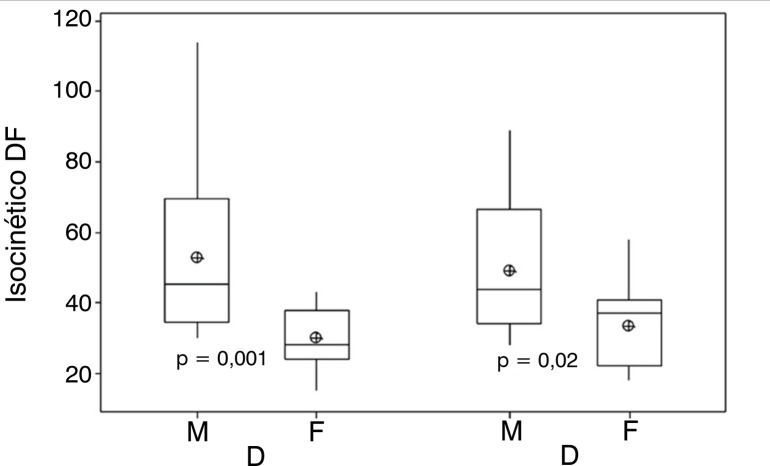
Comparison of the isokinetic DF strength of dominant (D) male (M) x female (F) and non-dominant (ND) male x female limbs. Legend: ⊕ = mean.

Figure 1 shows the isokinetic strength of DF comparing first the dominant limb between the male and female groups and then the non-dominant limb also between the male and female groups. It presents a significant difference between the variables studied.

**Figure 2:**
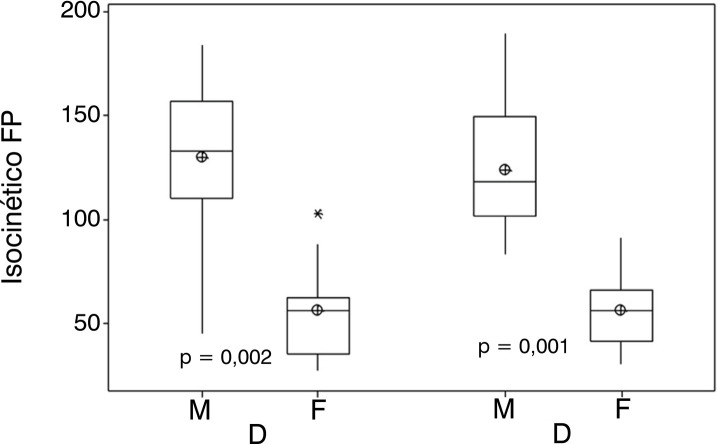
shows the isokinetic strength of PF comparing first the dominant limb between the male and female groups and then the non-dominant limb also between the male and female groups. It presents a significant difference between the variables studied. Legend: *: highest observed values; ⊕ = mean.

Figure 2 shows the isokinetic strength of PF comparing first the dominant limb between the male and female groups and then the non-dominant limb also between the male and female groups. It presents a significant difference between the variables studied.

**Figure 3:**
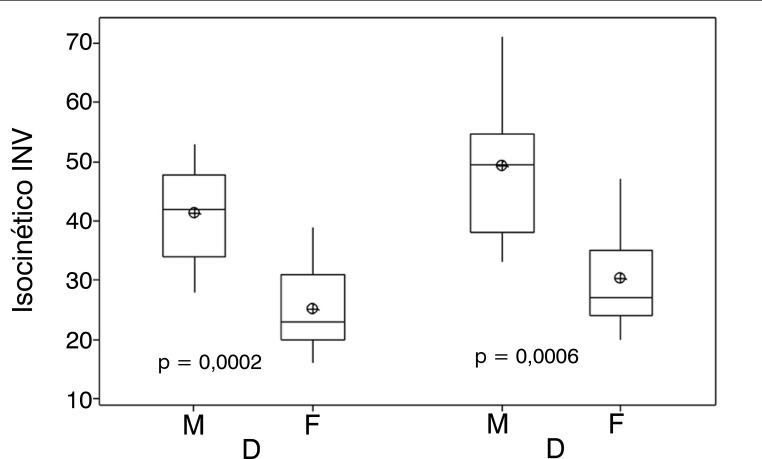
Shows the isokinetic strength of INV, first comparing the dominant limb between the male and female groups and then the non-dominant limb also between the male and female groups. It presents a significant difference between the variables studied. Legend: ⊕ = mean.

Figure 3 shows the isokinetic strength of INV, first comparing the dominant limb between the male and female groups and then the non-dominant limb also between the male and female groups. It presents a significant difference between the variables studied.

**Figure 4:**
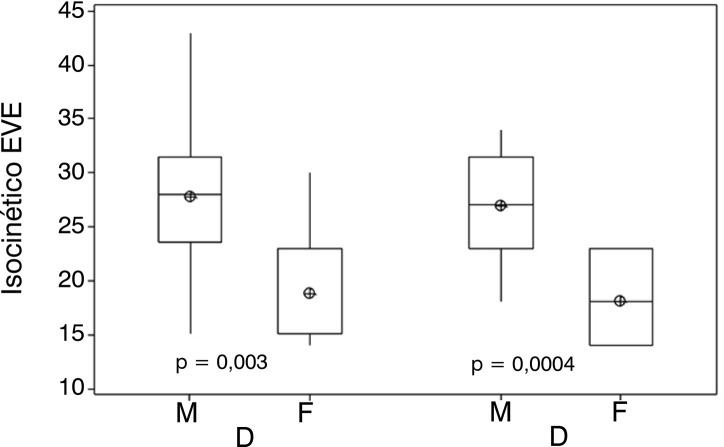
Shows the isokinetic strength of EVE comparing first the dominant limb between the male and female groups and then the non-dominant limb also between the male and female groups. It presents a significant difference between the variables studied. Legend: ⊕ = mean.

Figure 4 shows the isokinetic strength of EVE comparing first the dominant limb between the male and female groups and then the non-dominant limb also between the male and female groups. It presents a significant difference between the variables studied.

After analyzing the data, a significant difference in isokinetic strength was observed in which men had greater strength than women and very active individuals were stronger than irregularly active and sedentary individuals. Furthermore, the strength of the dominant side was greater than the non-dominant side.

On the other hand, analyzes related to isometric strength did not show significant differences between sex, dominance and level of physical activity.

## DISCUSSION

The present study was considered a pilot of large research that intends to associate variables of isometric and isokinetic strength in the ankle. We evaluated 25 healthy individuals of both genders and different levels of physical activity. With the preliminary results, significant differences were found between genders for dominant and non-dominant limbs in all isokinetic strength movements. In terms of isometric strength, no significant differences were found, possibly due to the current sample size.

There was a relevant difference between strength and physical activity levels. In the isokinetic strength of dominant and non-dominant plantar flexion, and isometric strength of non-dominant inversion, it was observed that individuals classified as very active presented a higher peak torque in relation to individuals classified as irregularly active and sedentary. This corroborates the results reported by Keles et. Al,^14^ who analyzed 24 healthy male recreational athletes divided into two groups. The control group did not practice exercises, and the exercise group practiced evertor and dorsiflexor activities. After six weeks, the exercise group demonstrated a significantly higher peak eccentric torque than the control group in dorsiflexion and eversion movements.^14^

The differences in strength between genders found in the present study is in agreement with the study by Preininger et. al, who analyzed 93 computed tomography scans of the pelvis of 45 men and 48 women. They observed greater muscle strength in men due to the greater absolute total volume of the hip muscular system (p<0.0001).^15^

Spink et al. investigated the isometric strength of inversion and eversion in individuals aged between 23.2 ± 4.3 years of both sexes, comparing the right and left sides, without taking into account limb dominance. For inversion force they demonstrated values of 18.8 ± 4.5 and for eversion force 18.2 ± 3.7.^16^

The sample of the present study was composed of subjects between 18 and 60 years old, subdivided by gender and dominance, in the analysis of the same variable. The isometric strength values of male subjects on the dominant side were 10.6 ± 4.3 and 13.1 ± 4.0 for inversion and eversion respectively, while the non-dominant side were 12.2 ± 5.5 and 12 .5 ± 3.9 of inversion and eversion respectively. Female individuals on the dominant side had 10.1 ± 4.2 and 13.3 ± 6.2 of inversion and eversion respectively, while the non-dominant side had 11.1 ± 4.2 and 12.1 ± 6 .3 of inversion and eversion respectively.

In the present study, the mean values of maximum isometric strength of the inversor and evertor muscles of the ankle were lower than the mean values found by Spink et al,^16^ who positioned the tested limb in hip and knee extension, which is a likely explanation for the difference between the results of both studies.

In this study, we found significant differences in isokinetic and isometric strength between men and women, as well as dominant and non-dominant side. Corroborating these data, Pellicer-Chenoll et al. compared the ratio between hamstrings and quadriceps, at different knee angles, to determine differences between gender and dominance. The ratio in the dominant limb demonstrated an average of 9% higher than that of the non-dominant limb. Another contribution of this study was in relation to limb positioning. The ratios were on average 53.4% lower in positions close to flexion than in positions close to extension.^17^ This piece of information supports the concern in maintaining the appropriate positioning of the two strength measurements, ensuring that both are like each other.

Kim et al. analyzed muscle strength in flexion and extension of the hip, knee and ankle using an isometric dynamometer in 55 students, 19 men and 36 women. The intra-examiner reliability found was above 0.9 for hip, knee and ankle flexion and extension strengths. Likewise, inter-rater reliability was found to be above 0.8 for the same measures. Therefore, the objective of the present study is viable and of high interest to the scientific community. Although there is a difference in the positioning of the volunteers, the strength values found are like previous studies.^18^

This study has limitations. Association, comparison and agreement calculations for the values were not performed, as the sample size was not considered sufficient to carry out these statistical tests. There are still few consistent studies, with robust methodology, that demonstrate normative data on foot and ankle strength, as well as other joints. ^19^ These data are essential for further investigation of the same variables in pathological conditions. Therefore, in the present study, the search for these results began in a group of healthy individuals. Future studies are needed to verify the correlation between ankle strength assessment methods.

## CONCLUSION

The results of the present pilot study demonstrated greater isokinetic strength in men than in women, as well as in active individuals compared to sedentary ones. However, it was not possible to establish an association between isometric and isokinetic ankle measurements.
